# Nicotinamide Mononucleotide Restores NAD^+^ Levels to Alleviate LPS-Induced Inflammation via the TLR4/NF-κB/MAPK Signaling Pathway in Mice Granulosa Cells

**DOI:** 10.3390/antiox14010039

**Published:** 2024-12-31

**Authors:** Mehboob Ahmed, Umair Riaz, Haimiao Lv, Muhammad Amjad, Sohail Ahmed, Shaokat Ali, Muhammad Usman Ghani, Guohua Hua, Liguo Yang

**Affiliations:** 1 Hubei Hongshan Laboratory, Wuhan 430070, China; 2 National Center for International Research on Animal Genetics, Breeding and Reproduction (NCIRAGBR), Ministry of Science and Technology, Huazhong Agricultural University, Wuhan 430070, China; 3 Department of Theriogenology, Faculty of Veterinary and Animal Sciences, The Islamia University of Bahawalpur, Bahawalpur 63100, Pakistan; 4 Key Laboratory of Agricultural Animal Genetics, Breeding and Reproduction of Ministry of Education, College of Animal Science and Technology, Huazhong Agricultural University, Wuhan 430070, China; 5 Medical Research Institute, Southwest University, Chongqing 400715, China

**Keywords:** granulosa cell, NMN, NAD^+^, inflammation, apoptosis, ROS, steroidogenesis

## Abstract

Inflammation disrupts the normal function of granulosa cells (GCs), which leads to ovarian dysfunction and fertility decline. Inflammatory conditions such as polycystic ovary syndrome (PCOS), primary ovarian insufficiency (POI), endometriosis, and age-related ovarian decline are often associated with chronic low-grade inflammation. Nicotinamide mononucleotide (NMN) is an important precursor of NAD^+^ and has gained attention for its potential to modulate cellular metabolism, redox homeostasis, and mitigate inflammation. This study investigated the protective roles of NMN against lipopolysaccharide LPS-mediated inflammation in GCs. The results of this experiment demonstrated that LPS had negative effects on GCs in term of reduced viability and proliferation rates and upregulated the production of pro-inflammatory cytokines, including interleukin-1 beta (IL-1β), interleukin-6 (IL-6), cyclooxygenase-2 (Cox-2), and tumor necrosis factor-alpha (TNF-α). Notably, the levels of NAD^+^ and NAD^+^/NADH ratio in GCs were reduced in response to inflammation. On the other hand, NMN supplementation restored the NAD^+^ levels and the NAD^+^/NADH ratio in GCs and significantly reduced the expression of pro-inflammatory markers at both mRNA and protein levels. It also enhanced cell viability and proliferation rates of GCs. Furthermore, NMN also reduced apoptosis rates in GCs by downregulating pro-apoptotic markers, including Caspase-3, Caspase-9, and Bax while upregulating anti-apoptotic marker Bcl-2. NMN supplementation significantly reduced reactive oxygen species ROS and improved steroidogenesis activity by restoring the estradiol (E2) and progesterone (P4) levels in LPS-treated GCs. Mechanistically, this study found that NMN suppressed the activation of the TLR4/NF-κB/MAPK signaling pathways in GCs, which regulates inflammatory processes. In conclusion, the findings of this study revealed that NMN has the potential to reduce LPS-mediated inflammatory changes in GCs by modulating NAD^+^ metabolism and inflammatory signaling pathways. NMN supplementation can be used as a potential therapeutic agent for ovarian inflammation and related fertility disorders.

## 1. Introduction

Inflammation is a well-known disruptor of ovarian function and has been associated with conditions such as impaired steroidogenesis, follicular atresia, and apoptosis. Inflammatory stimuli such as lipopolysaccharide (LPS) can trigger innate immune responses in GCs through pathways like Toll-like receptor 4 (TLR4) and result in increased production of pro-inflammatory cytokines. This inflammatory response has the potential to compromise the viability and functionality of GCs, which could lead to decreased steroidogenesis and altered follicular dynamics [1]. The link between ovarian dysfunctions and inflammation is evident across various conditions, such as polycystic ovary syndrome (PCOS), endometriosis, premature ovarian insufficiency (POI), and age-related ovarian decline [1,2]. Chronic inflammation not only disrupts normal ovarian function but also contributes to oxidative stress and cellular senescence, which ultimately reduces fertility and overall reproductive health. The published literature has also shown that inflammation is a major driver of NAD^+^ decline in cells [3,4,5]. A marked reduction in NAD^+^ levels within ovarian GCs under PCOS-like conditions has been observed [1]. Understanding these mechanisms highlights the importance of effectively addressing inflammation in managing ovarian disorders [6].

NAD^+^ plays a vital role in controlling energy metabolism in important reproductive events like folliculogenesis, oocyte maturation, and ovulation [1,7]. GCs provide critical nutrients and hormone signals and enable cellular communication, hence playing a substantial part in female fertility [8]. GCs often experience energy stress in the ovarian milieu as a result of continuous physiological events such as follicular development, atresia, and apoptosis [9,10]. As the follicle size increases, the energy demand rises, predominantly via glycolysis in GCs. Consequently, key substrates like pyruvate and lactate are produced to facilitate oocyte growth. In addition to its role in energy production, NAD^+^ is essential for genetic stability and supports vital processes such as DNA repair, cell proliferation, and tissue repair. It also plays a role in immune function, cellular senescence, and regulating inflammation [6,11,12]. A decline in NAD^+^ levels has been linked to reduced mitochondrial function and increased oxidative stress, both of which contribute to decreased GC viability and impaired oocyte quality [1]. Given its pivotal role in ovarian metabolism, it is not surprising that a decline in NAD^+^ is associated with diminished ovarian function.

The decline in NAD^+^ levels, which is linked with inflammation, intensifies oxidative stress and impairs normal cellular function, leading to increased apoptosis and a significant reduction in the quality of oocyte [13,14]. Therefore, it is crucial to maintain NAD^+^ homeostasis in GCs to maintain proper ovarian function and overall reproductive health. Gaining a better understanding of these mechanisms could open doors regarding therapeutic strategies to counter inflammation-related damage in the ovarian environment. Some recent studies have highlighted the therapeutic potential of NAD^+^ precursors, such as nicotinamide mononucleotide (NMN), in mitigating oxidative stress and inflammation. NMN supplementation has been demonstrated to restore intracellular levels of NAD^+^, alleviate inflammation and oxidative stress, and improve cellular functions across various tissues, including the liver, kidney, heart, and brain [5,15,16]. Although its effects on reproductive tissues remain largely unexplored, emerging evidence strongly suggests that NMN can alleviate inflammation-related damage in GCs.

In this study, we used LPS to induce inflammatory responses and to investigate the potential of NAD^+^ supplementation, specifically through NMN, as a therapeutic approach to mitigate inflammation-induced damage in GCs. This study aims to evaluate the impact of LPS-induced inflammation on the intracellular level of NAD^+^ in GCs. We also aim to assess the impact of NMN supplementation on steroidogenesis and cytokine expression and to elucidate the underlying mechanisms involved, mainly focusing on the TLR4/NF-κB/MAPK signaling pathways. By addressing these objectives, this research seeks to provide insights into the role of NAD^+^ in ovarian health and explore the therapeutic potential of NMN in reproductive medicine.

## 2. Materials and Methods

### 2.1. Management of Experimental Animals

We used Kunming mice of 3 weeks old (weighing approximately 10–15 g). The mice were supplied by and housed in the Animal Facility Center of Huazhong Agricultural University under controlled conditions. The housing temperature was kept around 21 ± 2 °C, and the light/dark cycle was 12 h. Mice were given with ad libitum access to standard chow and drinking water.

### 2.2. Ovarian Tissue Collection, Granulosa Cell Isolation, and Culture

Ovarian tissues were harvested from female mice under sterile conditions. After harvesting, the ovaries were washed in DMEM/F12 (SH30126, HyClone, Cytiva, Marlborough, MA, USA) and immediately processed for GC isolation. The GCs were isolated under the protocol as described earlier [8,17], with slight modifications. The granulosa cells (GCs) suspensions were collected and cultured in DMEM/F12 with 10% FBS (HyClone, Cytiva, USA) and 1% penicillin + streptomycin (Biosharp, Beijing, China, Cat#BL505A) in the required cell culture plates and incubated in a CO_2_ incubator with the temperature set at 37 °C. The culture medium of cells was replaced the next day (i.e., after 24 h) with fresh culture media. The purity of the GCs was confirmed as described in an earlier study [18].

### 2.3. Experimental Protocol

After an initial 48-h culture, when the GCs reached about 80–90% confluency, varying concentrations (0.1, 1, 5, 10, 20, and 100 μg/mL) of LPS (L970739, Macklin, Shanghai, China) were added to the culture media for 24 h. This was done to determine the appropriate concentration of LPS for future experiments. Based on the initial findings, 10 μg/mL of LPS was used for the subsequent experiments. Next, to figure out the appropriate concentration of NMN, the LPS-treated GCs were supplemented with different concentrations of 1, 10, 100, 500, 1000, and 2000 μM of NMN (B886299, Macklin Biochemical Technology Co., Ltd. Shanghai, China) in the culture medium. We performed all experiments in triplicate.

### 2.4. Cell Viability and Counting Analysis

Cell Counting Kit-8 (ATWA31031, Abbkine, Atlanta, GA, USA) was used to evaluate the GC viability. As per the guidelines of the manufacturer, GCs were seeded in 96-well cell culture plates. The cells were cultured under standard conditions. Following treatments with the designated concentrations of LPS and NMN, we added CCK-8 solution of 10 μL to each well, which already contained 90 μL of serum-free culture medium. The culture plates were incubated for 2 h in a CO₂ incubator with the temperature set at 37 °C. Next, a microplate reader (PerkinElmer, Waltham, MA, USA) was used to record absorbance at 450 nm, and this reading was used for further analysis. The results were used to determine the viability of the GCs. For cell counting, after treatment, GCs were harvested and counted using an automated cell counter (Bio-Rad Laboratories, Inc., TC20TM, Hercules, CA, USA), as described earlier [17].

### 2.5. Quantification of mRNA Through QRT–PCR

Following the manufacturer’s guidelines and using the AFTSpin Tissue/Cell Fast RNA Extraction Kit for Animals (RK30120, ABclonal, Woburn, MA, USA), total RNA was extracted from GCs. RNA concentration and purity were assessed post-extraction utilizing a spectrophotometer. Next, we used the FastKing gDNA Dispelling RT SuperMix (KR118, Tiangen Biotech, Beijing, China) for cDNA synthesis from RNA through reverse transcription using 1 μg of total RNA. Then, qRT-PCR was carried out using the 2 × Universal-Blue SYBR-Green qPCR Master Mix (G3326, Servicebio, Wuhan, China) on a real-time PCR machine (BioRad-CFX96, Bio-Rad, Hercules, CA, USA). Finally, the relative mRNA expression of target genes used in this study was determined using the 2(−ΔΔCT) method. The gene expression of GAPDH was used as the internal control. Primer-Premier 5.0 was utilized for the purpose of designing primer sequences. Supplementary Table S1 lists the target gene primer sequences utilized in this work.

### 2.6. Detection of Target Proteins Through Western Blot

Target proteins were detected by Western blot adopting standard protocols, as described earlier [8]. Briefly, RIPA lysis buffer containing PMSF, phosphatase inhibitor A solution (1%), and phosphatase inhibitor B solution (1%), supplied by Servicebio (Wuhan, China), was used for the lysis of GCs and, subsequently, for protein extraction. Equal concentrations of protein samples were separated by SDS-PAGE gel (10% and 12%) and transferred onto PVDF membranes (Immobilon-P, Milli-pore, Billerica, MA, USA) via electrophoresis. Membranes were blocked and incubated with primary antibodies overnight at 4 °C and then with HRP-conjugated secondary antibodies for 2 h at RT. After washing, membranes were developed using an ECL kit (Biosharp, Hefei, China) and imaged with a chemiluminescence system (Tanon, Shanghai, China). Bands were quantified using ImageJ software (version 1.54d, NIH, Bethesda, MD, USA) and normalized to GAPDH. The membranes were stripped using a stripping buffer (SL1342, Membrane Regeneration Solution/Stripping buffer, Coolaber, Beijing, China) according to the guidelines of the manufacturer.

### 2.7. Measurement of Intracellular NAD^+^ Levels

We used the NAD^+^/NADH WST-8 Method Detection Kit (S0175, Beyotime, Shanghai, China) for quantification of the intracellular levels of NAD^+^ and NADH. As per the manufacturer’s instructions, GCs were cultured in 6-well cell culture plates, and following treatment, the culture medium was discarded. Approximately 1.0 × 10⁶ cells were lysed with pre-chilled cell lysis buffer (200 μL) that was specific for NAD^+^/NADH. Then, centrifugation of the samples was performed at 10,000× *g* for 15 min at 4 °C. The lysate was pipetted and split into two portions: one was utilized for measuring the total NAD^+^/NADH concentration, while the other was for detecting the NADH levels alone. For the total NAD^+^/NADH measurement, 50–100 μL of the supernatant was placed in a tube, incubated at 60 °C for 30 min, and then centrifuged again at 10,000× *g* at 4 °C for 5 min. Afterward, 20 µL of the processed supernatant was pipetted into a 96-well flat bottom plate. For NADH quantification, 20 µL of supernatant was directly transferred to a 96-well plate. Subsequently, ethanol dehydrogenase working solution (90 µL) was added to each sample, and the plate was placed in an incubator with a temperature of 37 °C for 10 min. After incubation, 10 µL of color-developing reagent was added. Absorbance at 450 nm was recorded by an ELISA reader (the Spark multimode microplate reader, Tecan Group Ltd., Männedorf, Switzerland).

### 2.8. Cellular ROS Assay

The ROS levels of GCs were assessed by using a commercially available ROS Assay Kit (R252; Dojindo, Kumamoto, Japan) according to the manufacturer’s guidelines. Briefly, the cultured cells were washed with phosphate-buffered saline (PBS) and incubated for 30 min at 37 °C with 5% CO₂ in a working solution prepared by diluting 10× DCFH-DA (dichlorodihydrofluorescein diacetate) loading buffer at 1:10 in double-deionized water, followed by adding DCFH-DA dye at a ratio of 1:1000, resulting in a final dye concentration of 10 µmol. Finally, the cells were washed three times with PBS, and the fluorescence signal was detected using a fluorescence microscope (BZ-X700, KEYENCE, Osaka, Japan).

### 2.9. ELISA Assay for Detection of Hormonal Level

After the initial culture and required treatments of the GCs, culture media were collected from the Control, LPS, NMN, and LPS + NMN groups at 24 h. The culture media were centrifuged at 1000× *g* for 20 min to remove any debris, and the supernatant was transferred into clean tubes for hormone concentration analysis. The estradiol (E2) and progesterone (P4) concentrations were determined using mice-specific ELISA kits supplied by CUSABIO Biotechnology Co., Ltd. (Cat.# CSB-E05109m and CSB-E05104m, respectively, Shanghai, China), as per the guidelines of the manufacturer. The absorbance was measured at 450 nm using a microplate reader (the Spark multimode microplate reader, Tecan Group Ltd., Männedorf, Switzerland).

### 2.10. Statistical Analysis

GraphPad Prism software version 9.5.1 (GraphPad Inc., La Jolla, CA, USA) was used for the statistical analysis of the data. Data are reported as mean values from a minimum of three independent experiments ± standard error of the mean (SEM). Data were examined for significance of difference utilizing one-way ANOVA (multiple groups) or Student’s *t*-test (two groups). Results with *p* < 0.05 were deemed statistically significant.

## 3. Results

### 3.1. Optimal Concentration and Characterization of LPS-Induced Inflammation in Ovarian Granulosa Cells

**Figure 1:**
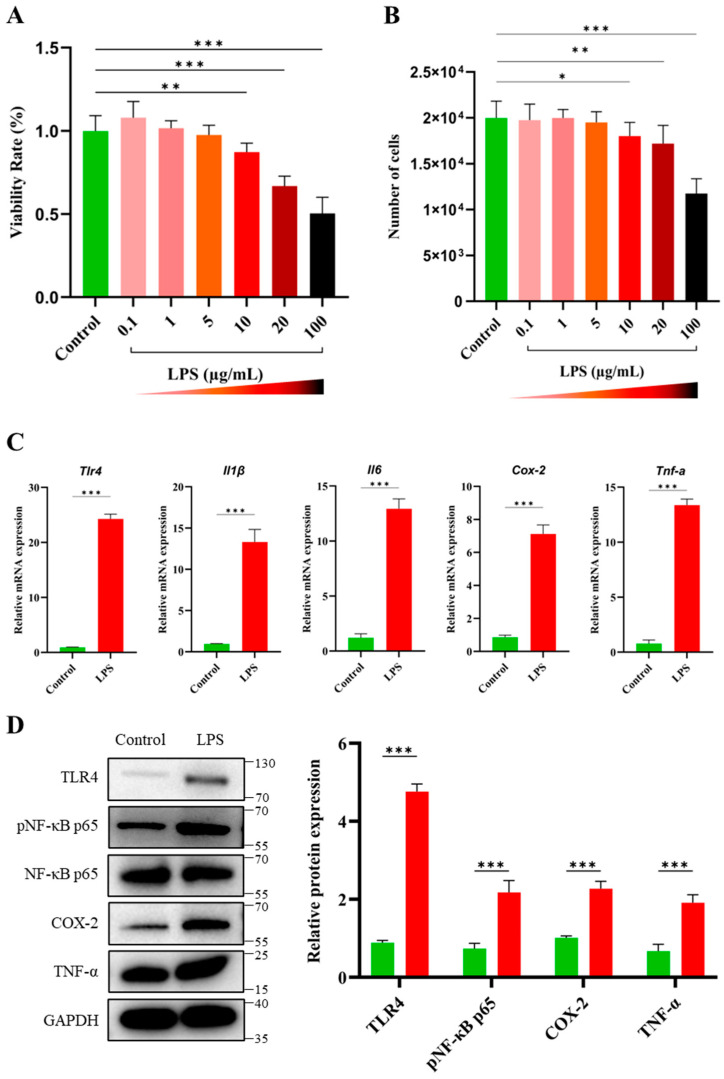
Effects of LPS on granulosa cells viability, proliferation, mRNA expression, and protein levels of pro-inflammatory markers. (**A**) Percentage of granulosa cell (GC) viability following treatment with varying concentrations of LPS (0.1, 1, 5, 10, 20, and 100 µg/mL) in culture media, showing a significant reduction at concentrations ≥ 10 µg/mL. (**B**) GC proliferation presented as the number of cells, with a significant decrease observed at 10 µg/mL LPS and higher. (**C**) mRNA expression of TLR4, IL-1β, IL-6, Cox-2, and TNF-α analyzed by qPCR, demonstrating significant upregulation in LPS-treated GCs compared to the control. (**D**) Western blot representative images and analysis showing increased protein expression of TLR4, phosphorylated NF-κB (p-NF-κB), COX-2, and TNF-α in response to LPS treatment, while GAPDH serves as the loading control. Values are expressed as mean ± SEM. Compared to the control group, * *p* < 0.05, ** *p* < 0.01, and *** *p* < 0.001.

LPS was administered at concentrations of 0.1, 1, 5, 10, 20, and 100 µg/mL in the culture media, and its effect on cell viability and proliferation was assessed. The results of this experiment demonstrated a concentration-dependent response. As shown in Figure 1A,B, no significant changes were observed at concentrations up to 5 µg/mL. However, for LPS concentrations of 10 µg/mL and higher (i.e., 20 and 100 µg/mL), there was a marked reduction in the cell viability and proliferation rates. At 10 µg/mL LPS concentration, a statistically significant decline in the GC viability rate and proliferation was observed (*p* < 0.01 and *p* < 0.05, respectively) compared to the control group. It indicated that this concentration significantly impaired cellular functions in terms of viability and proliferation. In light of these findings, 10 µg/mL LPS was chosen for further experiments to examine its effects on pro-inflammatory cytokine production.

Previous studies have shown that LPS interacts with Toll-like receptor 4 (TLR4), initiating a robust inflammatory response that leads to cytokine production. In this experiment, we first analyzed the gene expression of critical inflammatory markers in GCs following LPS treatment. The qPCR results showed a marked upregulation (*p* < 0.001) in the mRNA levels of TLR4, IL-1β, Il-6, Cox-2, and TNF-α in GCs in the LPS group as compared to the control (Figure 1C). These findings highlight that LPS significantly induced a pro-inflammatory transcriptional response in GCs. To further validate these transcriptional changes, Western blot analysis was performed, which demonstrated a remarkable upregulation (*p* < 0.001) in the protein expression of TLR4, phosphorylated NF-κB p65 (*p*-NF-κB p65), COX-2, and TNF-α in LPS-treated cells (Figure 1D). The increase in p-NF-κB p65 corroborates the upregulation of the NF-κB pathway, linking TLR4 stimulation to enhanced cytokine production. Collectively, these results demonstrated that LPS significantly upregulated both the transcription and translation of key inflammatory mediators, amplifying the inflammatory response in GCs.

### 3.2. LPS-Induced Inflammation Significantly Reduces the NAD^+^ Levels and NAD^+^/NADH Ratio in GCs

**Figure 2:**
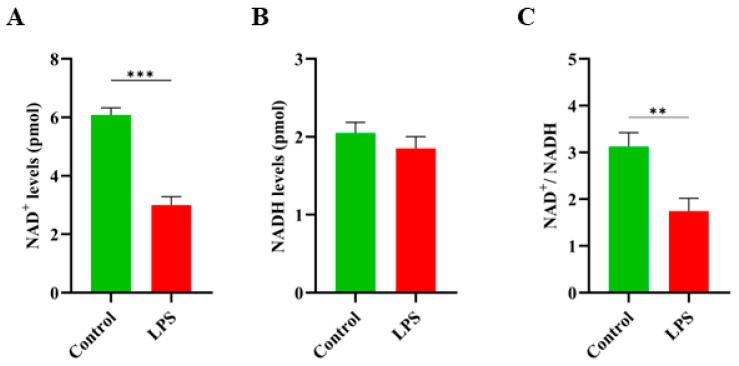
Effects of LPS on the NAD^+^ levels, NADH levels, and NAD^+^/NADH ratio in granulosa cells. (**A**) The NAD^+^ and NADH levels were measured in GCs (~1 × 10^6^ cells). (**A**) NAD^+^ levels in GCs after LPS treatment, showing a significant decrease compared to the control. (**B**) The NADH levels remained unchanged between the control and LPS-treated groups, indicating no significant effect of LPS on the NADH level. (**C**) The NAD^+^/NADH ratio in GCs, demonstrating a significant reduction in LPS-treated cells compared to the control. These data illustrate the metabolic shift induced by LPS in GCs, primarily driven by a reduction in the NAD^+^ levels and the NAD^+^/NADH ratio. Values are expressed as mean ± SEM. Compared to the control group, ** *p* < 0.01, and *** *p* < 0.001.

To investigate the effects of LPS-induced inflammation on cellular metabolism, we assessed the intracellular levels of NAD^+^ and NADH in GCs following LPS treatment. As depicted in Figure 2A, LPS treatment remarkably reduced the intracellular NAD^+^ concentration as compared to the control group (*p* < 0.001), indicating that the inflammatory milieu disrupts the cellular energy balance. Interestingly, the NADH levels remained unchanged (*p* ≥ 0.05) between the LPS-treated and control groups (Figure 2B), suggesting that LPS primarily impacts NAD^+^ metabolism without significantly affecting the NADH levels. Consequently, the NAD^+^/NADH ratio was significantly decreased in LPS-treated GCs, further supporting the metabolic shift induced by inflammation (Figure 2C). These findings suggest that LPS-mediated inflammation negatively impacts NAD^+^ metabolism, which may impair cellular function and viability.

### 3.3. Nicotinamide Mononucleotide (NMN) Improves Cell Viability and Proliferation Rate and Reduces the Pro-Inflammatory Cytokines Production in LPS-Treated GCs

**Figure 3:**
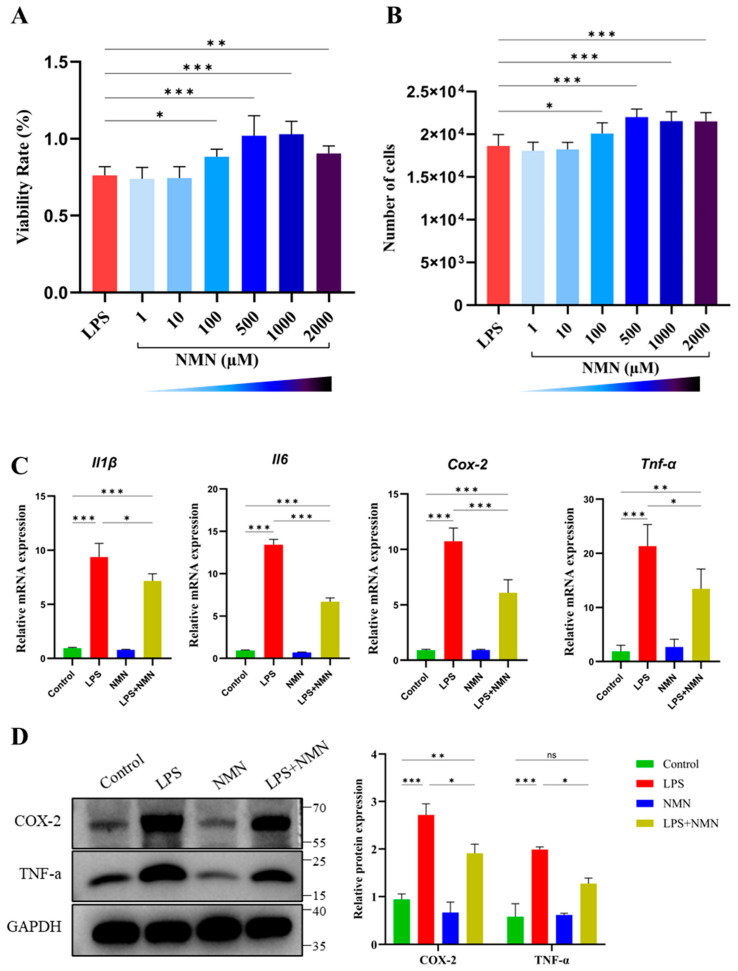
Effects of NMN supplementation on LPS-treated granulosa cells viability, proliferation, mRNA expression, and protein levels of pro-inflammatory markers. (**A**) Percentage of cell viability following treatment with varying concentrations of NMN (1, 10, 100, 500, 1000, and 2000 µM) in LPS-treated GCs, showing a significant increase at 100 µM, with the highest viability observed at 500 and 1000 µM. (**B**) GC proliferation presented as the number of cells, with a significant improvement observed at 100 µM and the highest rates observed at 500 and 1000 µM of NMN in LPS-treated GCs. (**C**) mRNA expression of IL-1β, IL-6, Cox-2, and TNF-α analyzed by qPCR, showing significant upregulation in LPS-treated GCs compared to the control, and a marked reduction in the LPS + NMN group as compared to LPS-treated GCs (**D**) Western blot representative images and analysis showing a reduced protein expression of Cox-2 and TNF-α in LPS + NMN-treated GCs compared to the LPS group. GAPDH was used as the loading control. Values are expressed as mean ± SEM. ns = non-significant; *p* ≥ 0.05, * *p* < 0.05, ** *p* < 0.01, and *** *p* < 0.001.

To determine the optimal concentration and the potential protective effects of NMN on LPS-treated GCs, different concentrations of NMN (1, 10, 100, 500, 1000, and 2000 µMol) were added to the culture media. The cell viability and proliferation rates were significantly high at 100 µM (*p* < 0.05), with the highest viability and proliferation rates obtained at 500 and 1000 µM of NMN (*p* < 0.001), compared to LPS-treated GCs without NMN supplementation (Figure 3A,B). Although viability was still higher at 2000 µM as compared to LPS-treated GCs without NMN supplementation, it was lower than at 500 and 1000 µM, suggesting a negative effect at higher concentrations. Based on these preliminary results, as well as the findings of earlier research that indicated higher doses of NMN may cause negative effects on tissues and cells due to local accumulation [19,20,21], we selected a concentration of 500 µM NMN for further experiments.

To further investigate whether NMN alleviates LPS-induced inflammation, mRNA expression of IL-1β, IL-6, Cox-2, and TNF-α was assessed through qPCR in four experimental groups: Control, LPS, NMN, and LPS + NMN. The results demonstrated that the mRNA expression of IL-1β, IL-6, Cox-2, and TNF-α was upregulated in the LPS group as compared to the control (*p* < 0.001). However, in the LPS + NMN group, NMN supplementation in the LPS-treated GCs remarkably reduced the expression of these pro-inflammatory markers compared to LPS treatment alone (Figure 3C). Of note, the NMN-alone treatment had no significant effect on cytokine expression compared to the control group (*p* ≥ 0.05), indicating that NMN alone does not impact GCs under normal conditions. However, the protective effect of NMN was most pronounced in the presence of LPS-induced inflammation. This suggests that NMN not only improves cell viability and proliferation but also effectively alleviates LPS-induced inflammation in LPS-treated GCs. These results were further validated at the protein level, which demonstrated that NMN supplementation remarkably reduced the protein expression levels of Cox-2 (*p* < 0.05) and TNF-α (*p* < 0.05) in LPS-treated GCs (Figure 3D). These findings are consistent with the anti-inflammatory effects observed at the mRNA level, confirming the role of NMN in mitigating inflammation.

### 3.4. NMN Increases NAD^+^ Levels and NAD^+^/NADH Ratio in LPS-Treated GCs

**Figure 4:**
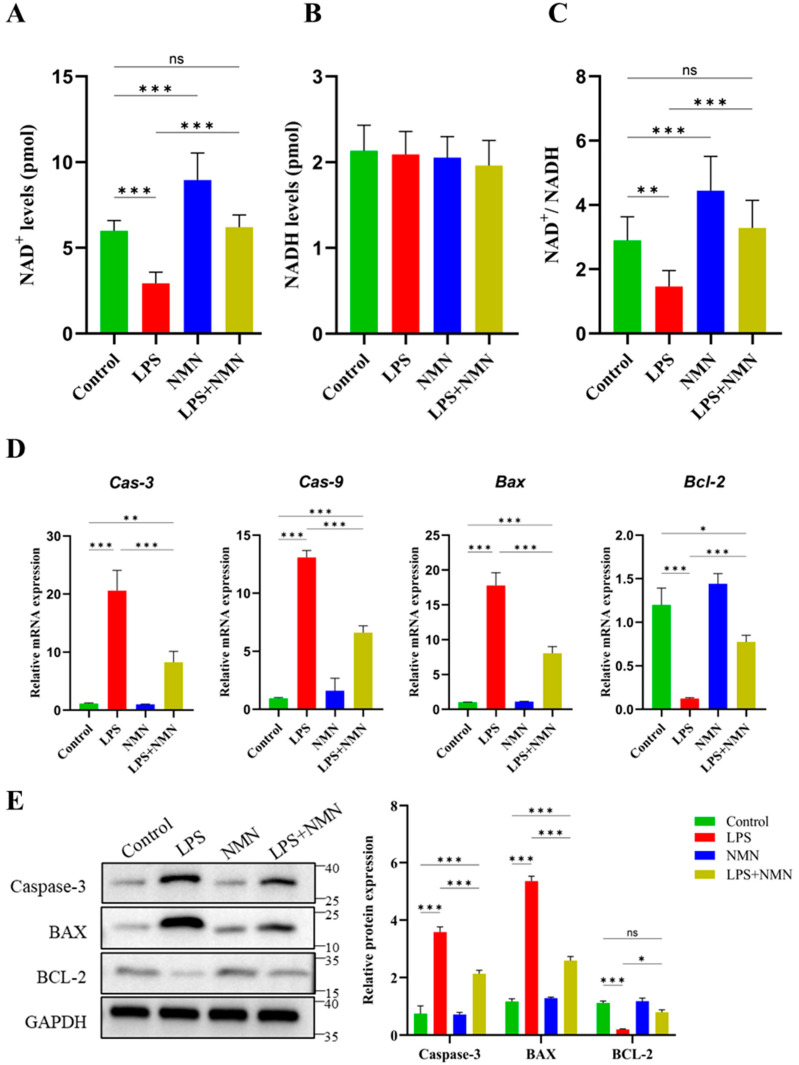
Effects of NMN supplementation on the NAD^+^ levels, NADH levels, and NAD^+^/NADH ratio in granulosa cells. The NAD^+^ and NADH levels were measured in GCs (~1 × 10^6^ cells) across four groups: Control, LPS, NMN, and LPS + NMN. (**A**) The NAD^+^ levels significantly decreased in LPS-treated GCs compared to the controls, while NMN supplementation restored the NAD^+^ levels to baseline. (**B**) The NADH levels showed no significant differences across the experimental groups. (**C**) The NAD^+^/NADH ratio, reduced by LPS treatment, was significantly restored with NMN supplementation. (**D**) The mRNA expression levels of apoptosis-related markers were assessed by qPCR. LPS treatment significantly upregulated the pro-apoptotic markers, including Caspase-3 (Cas-3), Caspase-9 (Cas-9), and Bax, while reducing the expression of the anti-apoptotic marker Bcl-2 compared to the control group. NMN supplementation (LPS + NMN) significantly reduced the expression of Cas-3, Cas-9, and Bax while restoring Bcl-2 expression in LPS-treated GCs. (**E**) Western blot analysis confirmed the protein-level changes in the apoptosis markers. LPS treatment increased the protein expression of Caspase-3 and Bax and reduced Bcl-2 expression compared to the control group. NMN supplementation significantly decreased the Caspase-3 and Bax levels and increased Bcl-2 protein expression in LPS-treated GCs. Data are presented as mean ± SEM with significance levels indicated: ns = non-significant; *p* ≥ 0.05, * *p* < 0.05, ** *p* < 0.01, and *** *p* < 0.001.

To evaluate the impact of NMN supplementation on NAD^+^ metabolism in LPS-treated GCs, intracellular levels of NAD^+^ and NADH were measured in four experimental groups: Control, LPS, NMN, and LPS + NMN. As shown in Figure 4A, a significant reduction in the NAD^+^ level was observed in LPS-treated GCs compared to the control group (*p* < 0.001). However, NMN supplementation in LPS-treated GCs (LPS + NMN) significantly increased the NAD^+^ levels as compared to the LPS group (*p* < 0.001), restoring levels to those comparable with the control group (*p* ≥ 0.05). It demonstrates that NMN supplementation has the ability to alleviate the inflammatory-induced decline in the NAD^+^ levels. Interestingly, the NAD^+^ levels were significantly higher in the NMN group compared to the control group (*p* < 0.001). However, no significant difference in NADH levels was observed across all the experimental groups, including control, LPS, NMN, and LPS + NMN (Figure 4B). It suggests that NADH metabolism remains unaffected by either LPS or NMN treatment. The NAD^+^/NADH ratio, a critical indicator of cellular energy balance and redox status, was significantly (*p* < 0.01) reduced in LPS-treated GCs compared to the control group (Figure 4C). In contrast, NMN supplementation in the LPS + NMN group markedly improved the NAD^+^/NADH ratio as compared to the LPS group (*p* < 0.001) and recovered it to levels similar to the control group (*p* ≥ 0.05). This demonstrated that NMN supplementation effectively restored the cellular energy balance disrupted by LPS-induced inflammation. Moreover, a significantly higher NAD^+^/NADH ratio was observed in the NMN group as compared to the control group (*p* < 0.001), suggesting that NMN supplementation enhances the metabolic efficiency in GCs even without inflammatory stimuli. Our results show that NMN supplementation significantly restored the NAD^+^ levels and the NAD^+^/NADH ratio in LPS-treated GCs. These findings underscore NMN supplementation’s broad protective and metabolic benefits in GCs.

### 3.5. NMN Alleviates the Apoptosis in LPS-Treated GCs

The expression of key apoptosis-related markers was analyzed at both the mRNA and protein levels to assess the effect of NMN supplementation. The LPS treatment significantly upregulated (*p* < 0.001) the gene expression of pro-apoptotic markers, including Caspase-3 (Cas-3), Caspase-9 (Cas-9), and Bax, as compared to the control group. In contrast, it reduced the expression of the anti-apoptotic marker Bcl-2 (*p* < 0.001) (Figure 4D). However, NMN supplementation in LPS-treated GCs (LPS + NMN) significantly (*p* < 0.001) reduced the mRNA expression of Cas-3, Cas-9, and Bax while restoring Bcl-2 expression. This indicates that NMN supplementation alleviates LPS-induced apoptosis by reducing the expression of apoptosis-related genes while enhancing the expression of anti-apoptotic genes.

Western blot analysis further validated these findings at the protein level. LPS treatment significantly increased the protein expression of Caspase-3 and BAX compared to the control group (*p* < 0.001), confirming the activation of the apoptotic pathway in LPS-treated GCs (Figure 4E). Furthermore, LPS treatment significantly reduced the expression of anti-apoptotic marker Bcl-2. However, NMN supplementation markedly reduced the protein levels of both Caspase-3 and BAX (*p* < 0.001) while increasing the protein expression of BCL-2 (*p* < 0.05) in the LPS + NMN group as compared to the LPS group. These results clearly reveal that NMN has the ability to alleviate LPS-induced apoptotic stress in GCs. NMN supplementation effectively counters LPS-induced apoptosis in GCs by downregulating pro-apoptotic markers and, at the same time, upregulating the anti-apoptotic marker.

### 3.6. NMN Reduces the ROS Levels in LPS-Treated GCs

**Figure 5:**
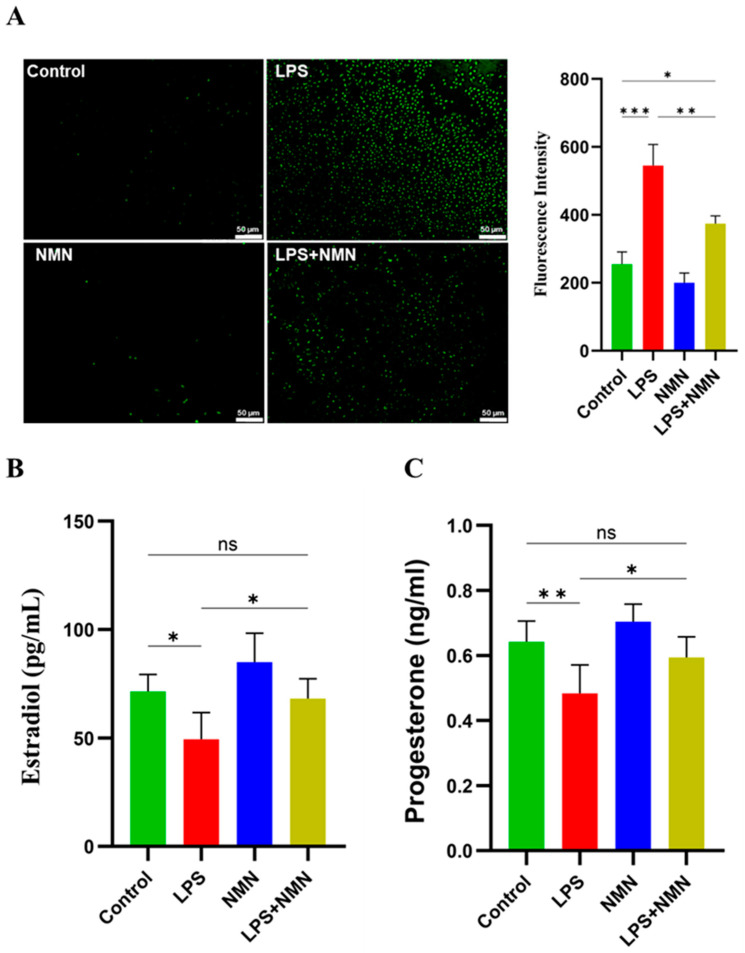
Effects of NMN supplementation on the ROS levels and steroidogenesis activity in granulosa cells. (**A**) Intracellular ROS levels were measured in GCs to assess oxidative stress. LPS-treated GCs showed a significant increase in ROS compared to the control group, while NMN supplementation reduced ROS in the LPS + NMN group, indicating protective effects against oxidative stress. (**B**,**C**) The levels of estradiol (E2) and progesterone (P4) in GCs were also evaluated. LPS treatment significantly reduced E2 and P4 production, whereas NMN supplementation restored their levels to the control values, supporting steroidogenic function. Values are presented as mean ± SEM, with significance levels indicated: ns = non-significant; *p* ≥ 0.05, * *p* < 0.05, ** *p* < 0.01, and *** *p* < 0.001.

The intracellular reactive oxygen species (ROS) levels were evaluated in GCs following LPS treatment. LPS-treated GCs exhibited a significant increase (*p* < 0.001) in the ROS levels as compared to the control group, as shown in Figure 5A. In contrast, NMN supplementation in LPS-treated GCs (LPS + NMN) resulted in a marked reduction in the ROS levels as compared to the LPS group (*p* < 0.01). These findings demonstrate that NMN has protective effects against oxidative damage in LPS-treated GCs. Notably, there was no significant difference in the ROS levels between the control group and the NMN group, which suggests that, under normal conditions, NMN has no effect on the ROS levels. These findings highlight the potential of NMN to mitigate LPS-induced oxidative stress in GCs, thereby supporting cellular health and function in inflammatory environments.

### 3.7. NMN Restores Steroidogenesis Activity in LPS-Treated GCs

To investigate the impact of LPS on steroidogenesis in GCs, the production of estradiol (E2) and progesterone (P4) was evaluated across the experimental groups. The results indicated that LPS treatment significantly reduced the E2 (*p* < 0.05), as well as P4, levels (*p* < 0.01), respectively, in GCs compared to the control, confirming that LPS exposure disrupts normal steroidogenic function (Figure 5B,C). However, NMN supplementation in the LPS-treated GCs (LPS + NMN group) effectively restored E2 and P4 production to levels comparable with the control group (*p* ≥ 0.05). These findings underscore the protective role of NMN in supporting steroidogenesis under inflammatory conditions, highlighting its potential to preserve the functional integrity of GCs by restoring E2 and P4 production.

### 3.8. NMN Alleviates LPS-Mediated Inflammatory Effect Through the TLR4/NF-κB p65/MAPK Pathway

**Figure 6:**
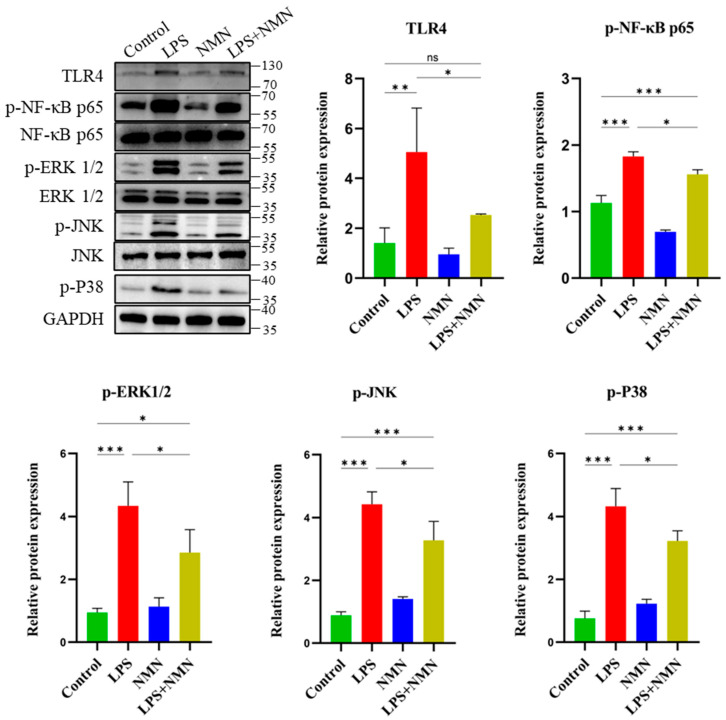
Effects of NMN on TLR4/NF-κB p65/MAPK pathway activation in granulosa cells. Western blot analysis shows that LPS treatment increased the expression of TLR4 (relative to GAPDH), p-NF-κB p65 (relative to NF-κB p65), p-ERK1/2 (relative to ERK1/2), p-JNK (relative to JNK), and p-P38 (relative to GAPDH) compared to the control group, while NMN supplementation in LPS-treated GCs reduced the expression of these proteins, indicating that NMN alleviates LPS-induced inflammatory signaling through the TLR4/NF-κB p65/MAPK pathway. Values are expressed as mean ± SEM; with significance levels indicated: ns = non-significant; *p* ≥ 0.05, * *p* < 0.05, ** *p* < 0.01, and *** *p* < 0.001.

To investigate the molecular mechanisms underlying through which NMN exhibits anti-inflammatory effects in LPS-treated GCs, we examined the involvement of the TLR4/NF-κB p65/MAPK signaling pathways. At the protein level, the Western blot analysis revealed that LPS-treated GCs showed a significant increase in the protein expression of TLR4 compared to the control group (*p* < 0.01), indicating activation of the TLR4-mediated inflammatory response (Figure 6). However, NMN supplementation in the LPS + NMN group markedly reduced the expression of TLR4 as compared to the LPS group (*p* < 0.05), demonstrating that NMN effectively attenuates LPS-induced activation of this pathway. Similarly, the protein expression of phosphorylated NF-κB p65 (p-NF-κB p65), a key mediator of inflammation, was significantly upregulated in LPS-treated GCs (*p* < 0.001) compared to the control, confirming LPS-induced activation of the NF-κB signaling pathway (Figure 6). In contrast, NMN supplementation significantly (*p* < 0.05) reduced the expression of p-NF-κB p65 in the LPS + NMN group as compared to the LPS, indicating that NMN mitigates LPS-induced NF-κB activation. Notably, the expression of p-NF-κB p65 was measured relative to the total NF-κB p65 levels. In the MAPK pathway, we observed a significant increase in the phosphorylation of ERK1/2 (p-ERK1/2), p-JNK, and p-P38 in LPS-treated GCs compared to the control (*p* < 0.001), suggesting that LPS activates the MAPK pathway as part of the inflammatory response. However, NMN supplementation in the LPS + NMN group significantly reduced the p-ERK1/2, p-JNK, and p-P38 levels as compared to the LPS group (*p* < 0.05), further indicating that NMN alleviates LPS-mediated inflammatory effects through the MAPK pathway. These results collectively indicated that NMN significantly downregulated the TLR4/NF-κB p65/MAPK signaling pathways in LPS-treated GCs, thereby mitigating the inflammatory response induced by LPS. This suggests that NMN exerts its anti-inflammatory effects by targeting key molecular mechanisms involved in inflammation.

## 4. Discussion

Inflammation and its downstream signaling effects are associated with the pathogenesis of GCs, which ultimately leads to ovarian dysfunction in females [22,23,24]. In the ovarian microenvironment, especially in GCs, one consequence of inflammation is increased follicular atresia, which triggers apoptosis in a significant number of cells. Hence, timely clearance of apoptotic cells is imperative for curbing inflammation. The mechanisms underlying these inflammatory effects and the biology of NAD^+^ under these conditions in GCs remain largely unexplored. Therefore, this study investigates how NAD^+^ plays a crucial role in maintaining GC viability and functionality under inflammatory conditions induced by LPS.

In the current study, we found that LPS significantly reduced the cell viability and proliferation rates coupled with an increase in pro-inflammatory cytokines, including IL-1β, IL-6, Cox-2, and TNF-α, at the mRNA and COX-2 and TNF-α at the protein levels (Figure 1). These cytokines are well known to disrupt ovarian function by creating an inflammatory milieu [22,25,26,27]. Next, we investigated how LPS-mediated inflammation affected the NAD^+^ levels and NAD^+^/NADH ratio in GCs. It was observed that the NAD^+^ levels and the NAD^+^/NADH ratio decreased in response to inflammation induced by LPS (Figure 2). This decrease suggests that redox balance and energy metabolism in GCs are negatively disrupted by inflammation, therefore impairing their viability and functionality. An earlier study has also found that the PCOS-related inflammatory environment in GCs causes a decrease in the NAD^+^ levels [1]. Recently, several reports have shown that aging has been linked with a progressive decline in NAD^+^ levels in different tissues [28]. In addition to reduced NAD^+^ levels, an important hallmark of aging is the rise of chronic, low-grade inflammation, a phenomenon often referred to as “inflammaging” [29,30]. Given these facts, it was hypothesized that inflammation might disrupt NAD^+^ homeostasis and, ultimately, dysfunctions in GCs. Inflammatory processes, particularly those involving cytokines and reactive oxygen species (ROS), have been shown to consume NAD^+^ through the activation of NAD^+^-dependent enzymes like poly(ADP-ribose) polymerases (PARPs) and sirtuins [31]. These enzymes are crucial for cellular responses to DNA damage and metabolic regulation but are also known to deplete NAD^+^ during inflammation. Moreover, studies have reported that NAD^+^ levels are reduced in inflammatory conditions affecting organs such as the brain, heart, lungs, and liver [32,33].

In the ovarian microenvironment, GCs continuously engage in energy-demanding processes, including follicular growth, atresia, and apoptosis. In cellular energy metabolism, NAD^+^ plays a critical role, as it facilitates the transfer of electrons and supports ATP production in key metabolic processes such as the tricarboxylic acid (TCA) cycle, glycolysis, and oxidative phosphorylation [34]. NAD^+^ and its reduced form, NADH, are core components in redox reactions and play an important role in cellular redox balance. NAD^+^ has garnered significant attention in recent years for its potential antioxidant, anti-inflammatory, and anti-aging properties [4,35,36]. The therapeutic potential of NAD^+^ supplementation has become an interesting area of research, as boosting NAD^+^ levels has been shown to alleviate inflammation and promote tissue repair. For instance, NAD^+^ precursors, such as nicotinamide riboside (NR) and nicotinamide mononucleotide (NMN), have demonstrated efficacy in reducing inflammation and improving metabolic function in various animal models and human trials [31,32,33]. Our study demonstrated the protective effects of NAD^+^ precursors such as NMN, which has not been extensively explored in ovarian inflammation models.

NMN is an important precursor and a key NAD^+^ intermediate. It is a product of the NAMPT reaction [37] and has a crucial role in maintaining the intracellular balance of the NAD^+^ content [38,39,40]. Two major pathways are involved in NMN synthesis: one involves nicotinamide (NAM) being converted by NAMPT, while the other uses nicotinamide riboside (NR), which is processed by NR kinases (NRKs) [41]. Recently, several reports have demonstrated that boosting NAD^+^ biosynthesis provides protective effects against conditions including diabetes, Alzheimer’s disease, endothelial dysfunction, and inflammation. This intervention may also mitigate age-related declines in NAD^+^ levels across multiple organs [5,20,38,42].

The results of this study demonstrate that NMN supplementation effectively restores NAD^+^ levels and enhances cell viability and proliferation rates in LPS-treated GCs (Figure 3 and Figure 4). The mRNA levels of inflammation-related genes (IL-1β, IL-6, Cox-2, and TNF-α) were significantly elevated in LPS-treated GCs. Conversely, NMN supplementation alleviated the effect of LPS-mediated inflammation by downregulating the above markers. Western blot analysis confirmed the reduction of Cox-2 and TNF-α at the protein level. These results are consistent with previous studies reporting that inflammation can cause a marked reduction in the intracellular levels of NAD^+^, whereas NAD^+^ supplementation can improve the health span in inflammation-linked diseases [43,44]. Prior studies have revealed the relationship between low levels of NAD^+^ with inflammation, as well as aging [43,45,46].

Additionally, in LPS-treated GCs, NMN supplementation reduced the mRNA expression of pro-apoptotic markers Caspase-3, Caspase-9, and Bax alongside an increase in the anti-apoptotic marker Bcl-2. These results were also further validated at the protein level by Western blot analysis (Figure 4). These results align with previous studies demonstrating the role of NAD^+^ and its precursors in regulating apoptosis across various cell types. For instance, NAD^+^ has been shown to prevent apoptosis in neutrophils and neurons by protecting the cells from oxidative, as well as chemical, stressors [11,47,48,49]. Additionally, the NAD^+^-dependent inhibition of SIRT1 in apoptotic signaling pathways suggests that NMN may offer broad anti-apoptotic benefits [3]. This underscores its potential therapeutic applications across diverse cellular environments.

Reactive oxygen species (ROS) are considered crucial mediators in cellular signaling and homeostasis, particularly in inflammatory disorders [50]. A significant factor in the pathogenesis is the chronic or prolonged production of ROS, and extensive research has focused on the role of ROS in various disease models [51,52,53]. Previous studies have shown that ROS acts as secondary messengers that amplify inflammatory signaling [54,55]. Studies have also shown that the reduction of NAD^+^ levels with aging is closely associated with the increased production of ROS, which is mediated by mitochondrial dysfunction and inflammatory processes [16]. Therefore, we explored whether intracellular NMN supplementation could reduce GC inflammation by attenuating ROS production. Our results showed that LPS significantly increased the level of ROS in GCs, while NMN supplementation increased the intracellular levels of NAD^+^, which significantly reduced the levels of ROS. The results align with prior research on the effects of NAD^+^ on ROS metabolism in inflammation-induced models [16].

We next evaluated the steroidogenesis capacity of GCs, which is considered crucial for ovarian function. It was observed that the steroidogenesis activity of GCs was impaired (decreased E2 and P4 concentrations) by LPS-induced inflammation (Figure 5). Previous studies have also established the detrimental impact of LPS on GCs, particularly through TLR4-mediated pathways, leading to decreased steroidogenesis and impaired oocyte development [56,57,58,59]. This reduction in E2 and P4 is linked to the downregulation of key steroidogenic enzymes such as CYP19A1 (P450arom), which converts androgens to estrogens, and CYP11A1, which plays a crucial role in progesterone production [1,60]. We found that NMN supplementation restored the levels of E2 and P4 in LPS-treated GCs, suggesting the role of NMN in mitigating endocrine disruption in inflammatory environments.

**Figure 7:**
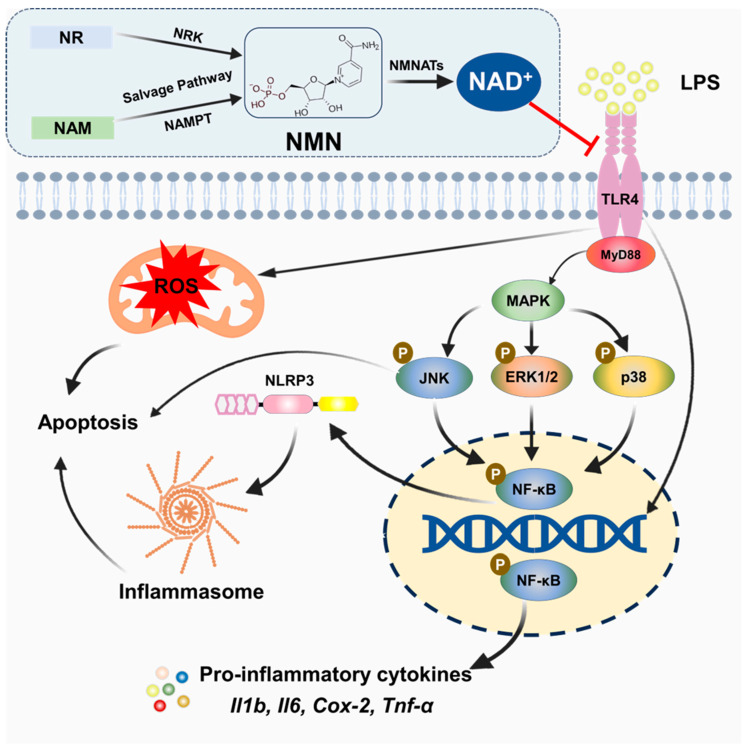
Graphical illustration of TLR4/NF-κB p65/MAPK pathway activation and inhibition by NMN. This illustration depicts the activation of the TLR4/NF-κB p65 and MAPK pathways in granulosa cells in response to LPS. LPS binding to TLR4 triggers downstream signaling, leading to NF-κB p65 phosphorylation and MAPK pathway activation, as indicated by the increased p-ERK1/2, p-JNK, and p-P38. The ROS levels and apoptosis are also increased in response to LPS-mediated TLR4 activation. NMN supplementation counteracts this activation by increasing the NAD+ levels and reducing TLR4 signaling and subsequently downregulating NF-κB p65 and MAPK phosphorylation, effectively mitigating the inflammatory responses, ROS production, and apoptosis markers. This graphical overview highlights the anti-inflammatory and anti-apoptotic roles of NMN and NAD+ in cellular signaling.

To investigate the mechanisms responsible for NMN-mediated therapeutic effects, we assessed TLR4/NF-κB/MAPK signaling pathways, which are generally induced by LPS and participate in inflammation, ROS, and apoptosis. The TLR4 signaling pathway is activated by many pathogen-associated molecular patterns (PAMPs), such as LPS. This pathway is considered an important mechanism for induced inflammation [61]. LPS-induced TLR4 signaling leads to the activation of various downstream pathways, including NF-κB [62,63], which translocates to the nucleus to initiate the production of pro-inflammatory cytokines such as TNF-α, IL-1β, and IL-6 [64,65,66]. In addition, another important mechanism of inflammation is the upregulation of the MAPK pathway by TLR4 signaling [65,67], which also promotes the production of pro-inflammatory factors by the phosphorylation of the ERK, JNK, and P38 proteins [61,67,68]. In this study, we found that NMN supplementation significantly downregulated these pathways, suggesting that NMN mitigates inflammation by inhibiting the TLR4-mediated signaling cascade, as illustrated in Figure 7. These findings are consistent with previous studies demonstrating that NAD^+^ boosting can alleviate inflammation through these pathways not only in ovarian models but also in cardiac, renal, and diabetic conditions [69,70,71,72]. By reducing cytokines production and apoptotic signaling, NMN appears to offer a protective mechanism against LPS-induced damage, contributing to ovarian health.

While our in vitro findings suggest that NMN may mitigate inflammation-induced ovarian dysfunction, future research should validate these effects in vivo, particularly in animal models of conditions like PCOS, premature ovarian failure, and endometriosis. Investigating the long-term impacts of NMN on fertility, hormonal regulation, and potential synergy with other anti-inflammatory agents will enhance its therapeutic applications in reproductive health.

## 5. Conclusions

In this study, we found that LPS-induced inflammatory effects were accompanied by a marked decline in NAD^+^ levels, highlighting the critical role of NAD^+^ in maintaining GCs function. NMN supplementation effectively restored the NAD^+^ levels in LPS-treated GCs, improving cell viability and proliferation while reducing the expression of pro-inflammatory markers, inhibiting apoptosis and ROS. Additionally, NMN enhanced steroidogenesis, alleviating the LPS-induced disruption in progesterone and estradiol production. Our findings suggest that NMN exerts its protective effects through the TLR4/NF-κB/MAPK signaling pathways, underscoring its potential as a therapeutic strategy for mitigating inflammation-induced ovarian dysfunction. These results not only provide insights into the role of NAD^+^ in ovarian inflammation but also suggest that NMN supplementation could have broader implications.

## Data Availability

The original contributions presented in this study are included in the article and Supplementary Materials. Further inquiries can be directed to the corresponding author.

## Supplementary Materials

The following supporting information can be downloaded at: https://www.mdpi.com/article/10.3390/antiox14010039/s1, Table S1: Primer information for qRT-PCR; Table S2: Antibodies information used for western blot.
