# Supplementary material for: Nicotinamide Mononucleotide Restores NAD+ Levels to Alleviate LPS-Induced Inflammation via the TLR4/NF-κB/MAPK Signaling Pathway in Mice Granulosa Cells
Source: Antioxidants (Basel). 2024 Dec 31;14(1):39. doi: 10.3390/antiox14010039 (PMC11762685; doi:10.3390/antiox14010039)
Supplement: Supplementary file 1 [file antioxidants-14-00039-s001.zip › antioxidants-3381298-supplementary.pdf]

Supplementary Table S1. Primer information for qRT-PCR

Primer Name	NCBI Accession Number	Primer sequence (forward)	Primer sequence (reverse)
Tlr4	NM_021297.3	TTCAGAGCCGTTGGTGTATC	CTCCCATTCCAGGTAGGTGT
Il1b	NM_008361.4	GCAACTGTTCCTGAACTCAACT	ATCTTTTGGGGTCCGTCAACT
Il6	NM_001314054.1	TAGTCCTTCCTACCCCAATTTCC	TTGGTCCTTAGCCACTCCTTC
Cox-2	NM_011198.5	TTCAACACACTCTATCACTGGC	AGAAGCGTTTGCGGTACTCAT
Tnf-α	NM_001278601.1	CCCTCACACTCAGATCATCTTCT	GCTACGACGTGGGCTACAG
Cas-3	NM_001284409.1	ATGGAGAACAACAAAACCTCAGT	TTGCTCCCATGTATGGTCTTTAC
Cas-9	NM_001277932.2	TCCTGGTACATCGAGACCTTG	AAGTCCCTTTCGCAGAAACAG
Bax	NM_001411994.1	TGAAGACAGGGGCCTTTTTG	AATTCGCCGGAGACACTCG
Bcl-2	NM_009741.5	GTCGCTACCGTCGTGACTTC	CAGACATGCACCTACCCAGC
Gapdh	NM_008084	TGACCTCAACTACATGGTCTACA	CTTCCCATTCTCGGCCTTG

Supplementary Table S2. Antibodies information used for western blot

Antibody name	Dilution	Supplier	Cat. No
TLR4	1:1000	Wanleibio, China	WL00196
p-NF-κB p65	1:500	Wanleibio, China	WL02169
NF-κB p65	1:1000	Wanleibio, China	WL01980
COX-2	1:1000	Wanleibio, China	WL01750
TNF-α	1:1000	Wanleibio, China	WL01581
Caspase-3	1:500	Wanleibio, China	WL04004
BAX	1:1000	Wanleibio, China	WL01637
BCL-2	1:500	Wanleibio, China	WL01556
ERK1/2	1:1000	Affinity Biologicals Inc. USA	AF0155
p-ERK1/2	1:2000	Cell signaling technology	4370S
JNK	1:500	Wanleibio, China	WL01295
p-JNK	1:500	Wanleibio, China	WL01813
p-P38	1:500	Wanleibio, China	WLP1576
GAPDH	1:3000	Servicebio Wuhan, China	GB15002
HRP-conjugated Goat anti-Mouse IgG	1:8000	ABclonal	AS003
HRP-conjugated Goat anti-Rabbit IgG	1:8000	ABclonal	AS014
